# Evaluation of Radiation Dose and Image Quality in Clinical Routine Protocols from Three Different CT Scanners

**DOI:** 10.3390/jimaging11030070

**Published:** 2025-02-25

**Authors:** Thawatchai Prabsattroo, Jiranthanin Phaorod, Piyaphat Tathuwan, Khanitta Tongluan, Puengjai Punikhom, Tongjit Maharantawong, Waraporn Sudchai

**Affiliations:** 1 Department of Radiology, Faculty of Medicine, Khon Kaen University, Khon Kaen 40002, Thailand; jiraph@kku.ac.th (J.P.); amponta@kkumail.com (P.T.); khanitta.th@kkumail.com (K.T.); puenpo@kku.ac.th (P.P.); tongma@kku.ac.th (T.M.); 2 Nuclear Technology Service Center, Thailand Institute of Nuclear Technology, Nakorn Nayok 26120, Thailand; newsudchai18@gmail.com

**Keywords:** radiation dose, image quality, high-contrast resolution, low-contrast detectability

## Abstract

Computed tomography examination plays a vital role in imaging and its use has rapidly increased in radiology diagnosis. This study aimed to assess radiation doses of routine CT protocols of the brain, chest, and abdomen in three different CT scanners, together with a qualitative image quality assessment. Methods: A picture archiving and communication system (PACS) and Radimetrics software version 3.4.2 retrospectively collected patients’ radiation doses. Radiation doses were recorded as the CTDI_vol_, dose length product, and effective dose. CT images were acquired using the Catphan700 phantom to evaluate image quality. Results: The findings revealed that median values for the CTDI_vol_ and DLP across the brain, chest, and abdomen protocols were lower than the national and international DRLs. Effective doses for brain, chest, and abdomen protocols were also below the median value of R. Smith-Bindman. Neusoft achieved higher spatial frequencies in brain protocols, while Siemens outperformed others in chest protocols. Neusoft consistently exhibited superior high-contrast resolution. Siemens and Neusoft outperformed low-contrast detectability, while Siemens also outperformed the contrast-to-noise ratio. In addition, Siemens had the lowest image noise in brain protocols and high uniformity in chest and abdomen protocols. Neusoft showed the lowest noise in chest and abdomen protocols and high uniformity in the brain protocol. The noise power spectrum revealed that Philips had the highest noise magnitude with different noise textures across protocols and scanners. Conclusions: This study provides a comprehensive evaluation of radiation doses and image quality for three different CT scanners using standard clinical protocols. Almost all CT protocols exhibited radiation doses below the DRLs and demonstrated varying image qualities across each protocol and scanner. Selecting the right CT scanner for each protocol is essential to ensure that the CT images exhibit the best quality among a wide range of CT machines. The MTF, HCR, LCD, CNR, NPS, noise, and uniformity are suitable parameters for evaluating and monitoring image quality.

## 1. Introduction

Computed tomography (CT) is a critical tool in diagnosing and screening diseases. According to a report by the United States of America, the cumulative effective dose received from medical procedures has increased due to the use of CT and is expected to continue to rise [1,2]. Therefore, controlling the quality and quantity of radiation is crucial to prevent excessive use. The principle of radiation protection is to use radiation as safely and effectively as possible, with the benefits outweighing the risks (justification), and use the minimum necessary amount of radiation for diagnosis (optimization) to prevent deterministic effects and reduce the risk of stochastic effects [3,4,5]. CT exams are high-radiation exams that require a careful consideration of the benefits and risks for each patient. It is essential for healthcare professionals to evaluate the need for a CT exam carefully and to use the lowest dose of radiation necessary to obtain the required diagnostic information.

Image quality evaluation in computed tomography should be assessed consecutively every year to ensure that image quality does not change when compared with the commissioning or recommended value. Many parameters determine image quality, such as high-contrast resolution, modulation transfer function, low-contrast resolution, the contrast-to-noise ratio, noise and uniformity, and CT number accuracy [6,7]. Many studies are concerned about image quality and radiation dose in clinical routine protocols and use CT scanners to monitor and ensure that the image quality remains [8,9,10]. Moreover, CT protocols in specific clinical indications could reduce patient radiation dose [11,12].

Although manufacturers provide specifications for radiation dose and image quality, there is a lack of systematic reviews of protocols and evaluations of image quality when using CT scanners over extended periods. Each institution encounters unique circumstances in producing CT images, including patient size, specific clinical indications, the performance of machines (whether old or new), radiologists’ experience and perception, and evolving reconstruction algorithms. These various factors affect protocol settings, radiation dose, image quality, and interpretation efficiency. All these elements are vital for optimizing protocols and ensuring that scanners operate effectively in clinical settings. In particular, in institutions with multiple CT scanners from different commercial brands, it is important to regularly monitor the performance, radiation dose, and image quality to maintain optimal imaging quality. Furthermore, this guideline has been essential in selecting appropriate CT scanners for each protocol, such as those for the brain, chest, or abdomen.

Therefore, the objectives of this study aimed to collect and compare the radiation dose from CT examination of patients in three different CT scanners from three clinical routine CT examinations of general indications in the brain, chest, and abdomen and quantitatively assess the image quality by using the Catphan700 phantom in the parameters of modulation transfer function (MTF), high-contrast resolution, low-contrast detectability (LCD), the contrast-to-noise ratio (CNR), image noise and uniformity and noise power spectrum (NPS). In addition, typical doses as median values were set to be compared to national or international diagnostic reference levels (DRLs) for radiation dose optimization while maintaining sufficient image quality in diagnosis. Moreover, decision making for selecting CT scanners in each clinical routine protocol was also determined for producing the best quality of CT images.

## 2. Materials and Methods

An institutional review board at the Center for Ethics in Human Research, Khon Kaen University, approved this study (HE631057). A total of 180 patients were collected retrospectively with a standard size (60 ± 15 kg) and ages between 18 and 80 years old. The radiation dose was collected from a picture archiving and communication system (PACS) and the Radimetrics radiation dose management platform using three clinical routine non-contrast phases, brain, chest, and abdomen, in three different CT scanners. The data were obtained by three different CT scanners, including SOMATOM Definition Flash (Siemens Healthineers, Erlangen, Germany) with 128 slices, Brilliance iCT (Philips Healthcare, OH, USA) with 128 slices, and Neusoft NeuViz 128 (Neusoft Medical Systems, Shenyang, China) with 128 slices.

### 2.1. Radiation Dose

The CTDI_vol_ and dose length product (DLP) were used as dose indices for CT and collected from PACS in the dose report and Radimetrics software version 3.4.2. The DLP is calculated by multiplying the CTDI_vol_ by the scan length, and the effective dose (ED) is calculated by multiplying the DLP by the conversion factor (k; mSv mGy^−1^ cm^−1^) for the head (0.0021), chest (0.014), and abdomen (0.015) which is specific to age and the region of scanning.

### 2.2. Image Acquisition Protocols

The scan parameters for all scanners are presented in Table 1. All CT scanners were performed with routine protocols, and all data acquisitions were performed thrice.

### 2.3. Image Quality Evaluation by Catphan 700 Phantom

A Catphan 700 phantom (The Phantom Laboratory Incorporated, Salem, NY, USA) was used to evaluate all image qualities. The Catphan 700 phantom acquired images with three CT scanners and three clinical routine protocols. The Auto QA Plus software version 1.8.6.0 (QA Benchmark, LLC, Frederick, MD, USA) was used to analyze image quality. Quality control (QC) testing was performed annually for all CT scanners, and the CT number was also calibrated before testing.

#### 2.3.1. The Modulation Transfer Function (MTF) and the High-Contrast Resolution (HCR)

The CTP682 module of the Catphan 700 phantom was employed to test the modulation transfer function (MTF) using wire tungsten with a 50 µm diameter. The MTF was determined by calculating the small wire tungsten’s point spread function (PSF). The PSF generates line spread functions (LSFs) in both vertical and horizontal directions. The MTF was calculated by taking the Fourier transform and presented in the value line pair/cm at 50%, 10%, and 2% of the MTF [8,13].

The CTP714 module assessed high-contrast resolution, encompassing resolution sections ranging from 1 to 30 line pairs per centimeter.

#### 2.3.2. Low-Contrast Detectability and Contrast-to-Noise Ratio (CNR)

Module CTP515 was used to determine the CNR, which contained low-contrast supra-slice targets with diameters of 15, 9, 8, 7, 6, 5, 4, 3 and 2 mm and three contrast levels of 0.3%, 0.5%, and 1.0%. TheCNR measured the difference between target signals and background signals. Low-contrast detectability was also calculated and shown as a theoretical contrast–detail curve. The curve showed the minimum contrast level at the given diameter that should be visible. CNR performance should meet the standards for 1 for adult head and abdomen protocol. Low-contrast detectability was compared among the CT scanners [9,13].

#### 2.3.3. Image Noise and Uniformity

CTP71 was used for the measurement of uniformity and image noise. Image uniformity was measured by using the difference values of the maximum HU of the center and the four peripheral ROIs at 3, 6, 9 and 12 o’clock locations. The noise level was defined as SD and measured at the center with an ROI diameter that was 40% of the phantom [8,9]. The difference between the mean CT value of each peripheral ROI and the center ROI should not exceed 5 HU [6].

#### 2.3.4. Noise Power Spectrum (NPS)

CTP71 was used to determine 2D-NPS, which used a single axial image for NPS processing. NPS determined the number of ROIs (16) and ROI size (64 pixels). Peak NPS frequency and frequency average were calculated and displayed as a graph of radial 1D-NPS [8,9].

#### 2.3.5. Statistical Analysis

Radiation doses, including the CTDIvol, DPL, and effective dose, are presented as mean ± SD, median and 75th percentile values. These values are then compared with those of national and international organizations’ values. Additionally, images obtained from three scans of the Cathan 700 Phantom were analyzed and expressed as mean ± SD. The parameters used for image quality evaluation were analyzed using AutoQA Plus software version 1.8.6.0 and compared with the reference and reference values provided by the American College of Radiology (ACR).

## 3. Results

### 3.1. Radiation Dose Evaluation in Computed Tomography

A total of 180 adult patients with a standard weight of 60 ± 15 kg and aged between 18 and 80 years old were collected in PACS and Radimetrics software version 3.4.2 to evaluate radiation dose according to the recommendation of ICRP Publication 135: Diagnostic Reference Levels in Medical Imaging [14]. The CTDIvol, DPL, and effective dose (ED) are standard dose metrics for evaluating and comparing CT scanners or protocols. The CTDIvol, DPL, and ED are shown in Table 2 in three clinical routine protocols for the brain, chest, and abdomen. The results were expressed as the mean ± SD, median, and 75th percentile. Median values were used to compare the DRLs of national and international organizations. It was found that the CTDIvol and DLP of the three protocols of the three scanners showed median values lower than Thailand DRLs 2021, Japan DRLs 2020, ACR DRLs 2018, and ICRP DRLs 2001 (Table 3 and Table 4). The three scanners’ effective dose of brain and chest protocols showed median values lower than those of the effective dose from R. Smith-Bindman and Ioannis Pantos (Table 5). While abdomen protocols for all protocols showed a lower value than the R. Smith-Bindman effective dose, the abdomen protocol for Neusoft showed a higher value than the Ioannis Pantos effective dose.

When comparing different CT scanners, the median values of the CTDI_vol_, DLP, and effective dose for Philips and Neusoft were found to be lower than those of another scanner in brain protocols. At the same time, Siemens and Philips exhibited lower CTDI_vol_, DLP, and effective dose values in the chest and abdomen protocols compared to Siemens.

### 3.2. Image Quality Evaluation

Image quality was evaluated using the quantitative analysis method via a Catphan 700 phantom. The three-time image scanning was obtained from three clinical routine protocols with three CT scanners from various manufacturers. AutoQA Plus software (QA Benchmark, LLC) version 1.8.9.0 was used to analyze and evaluate image quality.

#### 3.2.1. Modulation Transfer Function (MTF)

**Figure 1:**
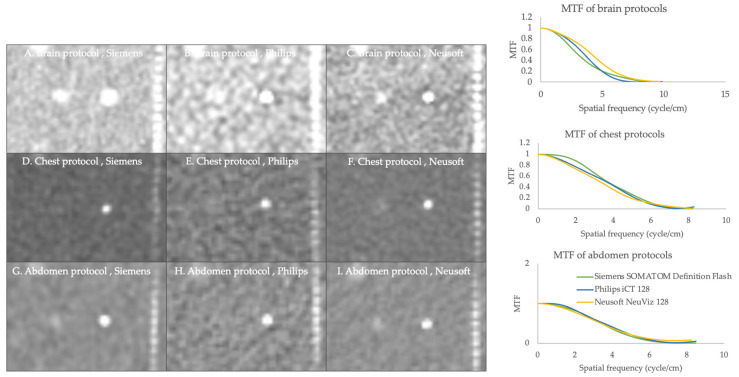
A comparison of the modulation transfer function of brain, chest, and abdomen protocols with various CT scanners. The small wire (indicated by the white dot in the axial image) is used to calculate MTF. The green, blue, and yellow lines represent the Siemens SOMATOM Definition Flash, Philips iCT128, and Nuesoft NeuViz 128, respectively.

The results presented in Table 6 and Figure 1 indicate that the modulation transfer function (MTF) varied across protocols and CT scanners. For instance, the Neusoft CT scanner (Neusoft Medical Systems, Shenyang, China) demonstrated higher spatial frequencies than other CT scanners in the brain protocol. Notably, Siemens’ chest protocol outperformed a higher frequency of 0.5 MTF compared to other CT scanners. However, the MTF of 0.1 and 0.02 exhibited similar frequencies in the chest protocol. Additionally, the abdominal protocols of all CT scanners exhibited similar spatial frequency patterns.

#### 3.2.2. High-Contrast Spatial Resolution

**Figure 2:**
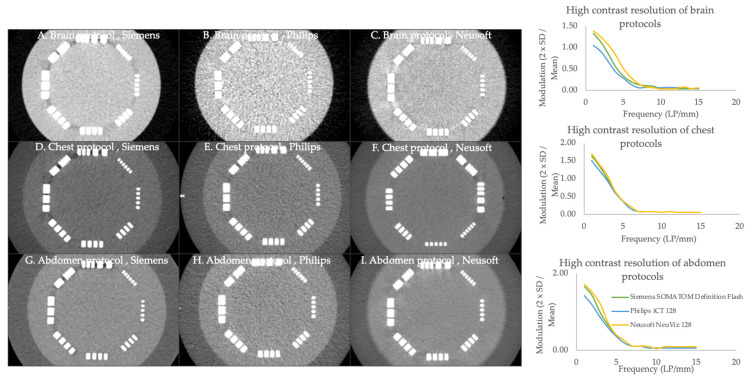
Comparison of high-contrast spatial resolution of brain, chest, and abdomen protocols with various CT scanners. The line pair (shown in the white line) is used to analyze high-contrast spatial resolution. The green, blue, and yellow lines represent the Siemens SOMATOM Definition Flash, Philips iCT128, and Nuesoft NeuViz 128, respectively.

The high-contrast resolution is illustrated in Figure 2. In the brain protocol, the Nuesoft CT scanner demonstrated superior performance at low frequencies, diminishing differences among the scanners at higher frequencies. Similarly, in the chest protocol, the Siemens and Neusoft scanners exhibited slightly higher modulation at lower frequencies than the Philips scanner. At the same time, all systems performed comparably at higher frequencies. In the abdomen protocol, the Neusoft and Siemens scanners marginally outperformed the other scanners at low to intermediate frequencies.

#### 3.2.3. Low-Contrast Detectability (LCD)

**Figure 3:**
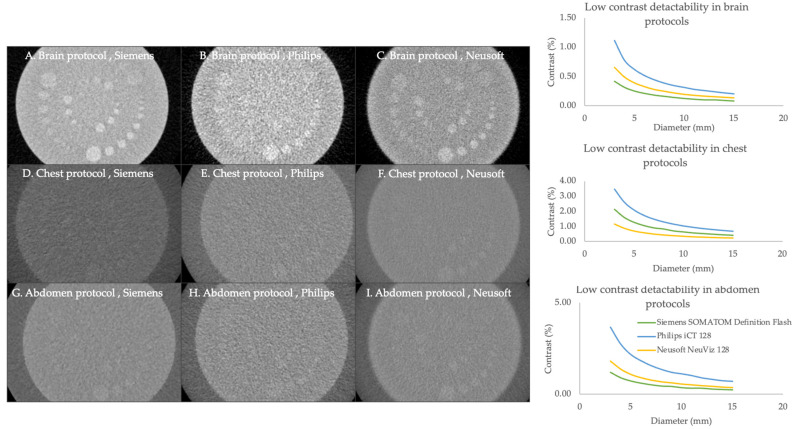
Comparison of low-contrast detectability of brain, chest, and abdomen protocols with various CT scanners. The cylindrical object at various diameter (shown in the white cylindrical dot) is used to analyze low contrast detectability and contrast-to-noise ratio. The green, blue, and yellow lines represent the Siemens SOMATOM Definition Flash, Philips iCT128, and Nuesoft NeuViz 128, respectively.

LCD is shown in Figure 3. In the brain and abdomen protocols, Siemens consistently exhibited superior detectability at the lowest contrast in the 3 mm diameter of the object, followed by Neusoft and Philips, while the Neusoft scanner performed better than the other CT scanners in chest protocol, which detected the smallest object of 3 mm in the lowest percentage contrast.

#### 3.2.4. Contrast-to-Noise Ratio

**Figure 4:**
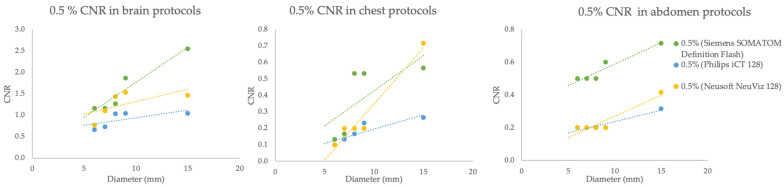
Comparison of contrast-to-noise ratio of brain, chest, and abdomen protocols with various CT scanners. The green, blue, and yellow lines represent the Siemens SOMATOM Definition Flash, Philips iCT128, and Nuesoft NeuViz 128, respectively.

The contrast-to-noise ratio (CNR) for a 0.5% contrast object is presented in Figure 4. Siemens demonstrated the highest CNR across a wide range of object diameters. Neusoft exhibited moderate performance, while Philips consistently showed the lowest CNR in all the protocols.

#### 3.2.5. Image Noise and Uniformity

The image noise and uniformity are summarized in Table 7. The image noise of the brain protocols for the Siemens CT scanner exhibited the lowest noise levels among all CT scanners, while Neusoft demonstrated a moderate noise level. Neusoft showed the lowest noise levels in the chest and abdomen protocols compared to all other CT scanners. Regarding uniformity, Neusoft achieved the best performance in the brain protocols, whereas Siemens exhibited superior uniformity in both the chest and abdomen protocols; moreover, it showed values within the range of the acceptable value (≤5 HU) [21].

#### 3.2.6. Noise Power Spectrum (NPS)

**Figure 5:**
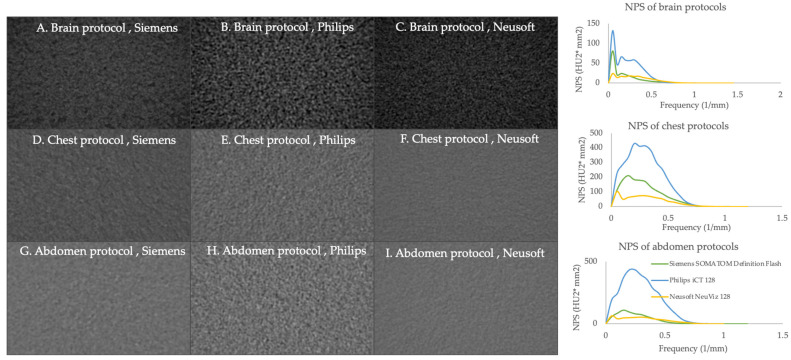
Comparison of noise power spectrum of brain, chest, and abdomen protocols with various CT scanners. The green, blue, and yellow lines represent the Siemens SOMATOM Definition Flash, Philips iCT128, and Nuesoft NeuViz 128, respectively.

The noise power spectrum (NPS) provides a comprehensive description of the noise properties by measuring the magnitude and spatial correlation of noise, often called texture (Figure 5). In the brain, chest, and abdomen protocols, the Philips CT scanners demonstrated the highest noise magnitude compared to Siemens and Neusoft. Table 8 shows the peak NPS frequency and average frequency, which indicates the noise texture of an image. The NPS curves show a different texture shape between the three CT scanners with various protocols. It was found that Philips CT scanners exhibited the highest peak NPS frequency in the chest and abdomen protocol, while the brain protocols showed the same peak NPS frequency. The average frequency in Siemens exhibited the lowest frequency in all protocols and scanners.

## 4. Discussion

This was the first study of our institute to provide a comprehensive evaluation of radiation doses and image quality across three different CT scanners in three routine clinical protocols by quantitative analysis via the Catphan 700 phantom. The result presented in-depth physical characteristics of CT images in each CT scanner. All protocols were carried out over many years without systematic reviews or image quality evaluations. The CTDI_vol_ and DLP were used as the standard dose metrics for computed tomography according to the recommendation of the ACR and guidelines from the ICRP Publication 135 [14], and they can be used to compare CT scanners. Moreover, the effective dose was also calculated to estimate radiation risk for CT examination. The findings indicate that the median values for the CTDIvol and DLP across brain, chest, and abdomen protocols were lower than the diagnostic reference levels (DRLs) established by national and international organizations, including Thailand 2021, Japan 2020, the ACR 2018, and the ICRP 2001. The three scanners’ effective dose of brain and chest protocols showed median values lower than the median value from R. Smith-Bindman and Ioannis Pantos. The abdomen of all scanners showed an effective dose lower than that in R. Smith-Bindman’s study, except for Neusoft CT, which showed a higher effective dose than that in Ioannis Pantos’ study. This suggests that the scanners employed operate within safe limits for radiation exposure, which is crucial for minimizing potential patient risks during imaging procedures. The CTDI_vol_ of this study showed values lower than the notification values recommended by the AAPM at 80 mGy and 50 mGy for adult brain and torso protocols, respectively [22].

Quantitative evaluation was used to evaluate the image quality of three CT scanners and three clinical routine protocols, which was easy, valid, and reliable for the preliminary assessment of CT imaging. The scanners and protocols showed different image qualities, described as the modulation transfer function (MTF), high-contrast resolution (HCR), low-contrast detectability (LCD), contrast-to-noise ratio (CNR), image noise and uniformity, and noise power spectrum (NPS).

The MTF quantifies the extent to which an imaging system can effectively transfer spatial frequencies from the object being imaged to the resulting final image. The Siemens and Neusoft scanners showed better MTF when compared to other CT scanners. However, all CT scanners and protocols met or exceeded the ACR recommendation values. According to the ACR recommendation, the limiting resolution must meet or exceed five cycles/cm and six cycles/cm for adult abdomen protocols and chest high-resolution CT, respectively [7]. From previous studies, the MTF values at 50% and 10% were 3–4 cycles/cm and 6–7 cycles/cm [9,13], respectively, which were similar to the values in this study. A higher frequency at a given MTF value indicates better image quality and resolution. High MTF values indicate good spatial resolution and detail preservation, especially in bone regions, contrast-filled areas, vessels, and small lesions. HCR refers to the ability of a CT scanner to distinguish between objects with a significant difference in X-ray attenuation, such as bone versus soft tissue. It is crucial for identifying small lesions, fractures, and other abnormalities. The pattern of HCR is similar among all CT scanners, and Neusoft and Siemens showed better HCR in all CT routine protocols.

LCD refers to the ability of a CT scanner to distinguish between objects with similar background attenuation coefficients. The LCD of Siemens consistently outperformed other scanners across brain and abdomen protocols at 0.4% contrast with 3 mm of rod and 1.2% contrast with 3 mm of rod, respectively. Meanwhile, Neusoft performed better in the chest protocol at 1.1% contrast with 3 mm of rods. According to the ACR’s recommendation, all four cylinders must be clearly visible at a 0.6% contrast with 6 mm rods [7]. In Luca Bellesi et al.’s study, LCD at 0.5% contrast showed the visibility of the rod ranging from 2 to 6 mm at various doses and reconstruction algorithms [13]. The capability of LCD is crucial for identifying subtle lesions or abnormalities that may not have a significant difference in density compared to surrounding tissues.

The CNR quantifies the ability to distinguish between an object of interest and its background based on the contrast in signal intensity relative to the noise level present in the image. In this study, The CNR findings revealed that Siemens for all protocols achieved the highest values across all object diameters, which is critical for ensuring clear images with minimal noise interference. At 0.5% contrast object with 6 mm rods, the CNR of the Siemens scanner was 1.3, 0.3, and 0.8 in the brain, chest, and abdomen protocols, respectively. According to the ACR recommendation, the CNR of the adult head and abdomen should meet 1.0 [6]. These findings underscore the significance of choosing CT systems that align with specific clinical needs, especially when a high contrast-to-noise ratio (CNR) is crucial for accurate diagnosis.

Image noise in computed tomography refers to unwanted variations in pixel values that disrupt the uniformity of an otherwise homogeneous image. This can cause a grainy appearance, obscuring important diagnostic information. On the other hand, uniformity in CT refers to the consistency of pixel values across a homogeneous region of an image. Ideally, a uniform area should display minimal variation in pixel intensity, suggesting that the imaging system functions correctly without significant noise interference. Patrizio Barca et al. showed that the noise values ranged from 3.5 HU to 5.7 HU across scanners and protocols [9]. In contrast, ACR does not determine the noise value because it depends on examination protocol and clinical indication. It should be compared with the commissioning value or determined by qualified medical physicists. Meanwhile, uniformity should not exceed 5 HU, and the center of HU should be within ±7 HU as ACR mentioned [7]. The study of Luca Bellesi et al. showed uniformity of approximately 0.5–2.5 HU [13]. CT Siemens scanners exhibited the lowest noise levels in brain protocols and the highest uniformity in chest and abdomen protocols. At the same time, Neusoft showed lower noise levels in chest and abdomen protocols and the highest uniformity in brain protocols. This variation in noise performance underscores the importance of selecting appropriate CT systems based on specific clinical needs.

NPS provides a comprehensive representation of how noise varies across different spatial frequencies. NPS provided insights into noise characteristics and texture among different protocols and scanners. Peak NPS frequency and average frequency determined the noise texture in CT images, in which higher peak NPS frequency and average frequency showed the finer grain texture of the image. In comparison, lower peak NPS frequency and average frequency showed coarser and grainier images. According to the study of Patrizio Barca et al., the result showed that the peak position of NPS curves ranged from 0.21 to 0.30 mm^−1^ [9] in CT head protocol, and the study by J. Greffier et al. showed that NPS spatial frequency ranged from 0.08 to 0.31 mm^−1^ at various algorithm and radiation dose [23]. Compared with this study, it was similar in noise level and texture. Understanding these noise magnitude and noise texture is essential for optimizing imaging techniques to enhance diagnostics performance.

This study has several limitations. First, using the Catphan700 phantom limits the clinical relevance of our findings as it lacks the anatomical complexity and variability found in patient images. However, we employed a phantom-based approach to ensure standardized and reproducible measurements of radiation dose and image quality while avoiding radiation exposure to human subjects. Future research should investigate the correlation between phantom-based measurements and image quality in clinical images to validate these findings and assess their applicability in a clinical setting. Second, we used standard clinical protocols without considering specific clinical needs or indications, which may affect the generalizability of the findings. Optimizing protocols for specific clinical scenarios could provide more tailored insights. Therefore, future studies should explore the impact of protocol optimization on radiation dose and image quality for specific clinical indications, such as stroke imaging or lung nodule detection. Third, the lack of qualitative evaluation by radiologists limits the assessment of image quality in clinical diagnostics. Incorporating expert evaluations would enhance the applicability of the study. Future studies should include expert evaluations by radiologists to assess the diagnostic acceptability of the images and correlate quantitative measurements with subjective image quality scoring. Finally, the study’s scope was limited to three CT scanners, and the data were not publicly available, which restricts the broader applicability and reproducibility of the findings. Future studies should include a wider range of CT scanners and consider making their data publicly accessible to enhance the generalizability and reproducibility of the findings. Despite these limitations, our study offers valuable insights into the relative performance of these three CT scanners using standardized protocols and quantitative image quality metrics. This study did not determine the good or bad performance of each CT scanner in commercial brands because clinical routine protocols were specific to each institute. They were different in CT technologies, software, algorithms, instruments, and exposure parameters, meaning that all of them can produce different image qualities and radiation doses. This study emphasized the in-depth physical characteristics of CT images to serve as a guideline for optimizing clinical protocols and appropriately selecting CT scanners in each clinical protocol.

## 5. Conclusions

This study provides a comprehensive evaluation of the radiation doses and image quality of three different CT scanners using standard clinical protocols. Almost all CT protocols exhibited radiation doses below the DRLs and demonstrated varying image qualities across each protocol and scanner, including in-depth physical characteristics of CT images. Choosing the appropriate CT scanner for each protocol is essential to ensure that the resulting CT images uphold the highest quality standards across various CT machines. Several key parameters, including the MTF, HCR, LCD, CNR, NPS, noise, and uniformity, are vital in assessing and monitoring image quality. Furthermore, our findings suggest that Siemens may be the preferred choice for all CT protocols due to its superior MTF, HCR, LCD, CNR, and noise performance, although the CTDIvol in the brain protocol was relatively high, which can be optimized to reduce the radiation dose. Alternatively, Neusoft was suitable for the chest protocol due to its good CNR and LCD and lower noise levels. Philips showed dominance regarding low-dose CT protocols, which are appropriate for patients who are children. Philips should be optimized by increasing radiation for improving LCD, the CNR, and noise. Monitoring radiation dose and image quality with DRLs, as well as among the CT scanners, ensures patient safety while achieving an appropriate radiation dose and maintaining sufficient image quality for interpretation.

## Figures and Tables

**Table 1:** Scan parameters for all scanners and protocols.

Parameters	Brain			Chest			Abdomen		
	Siemens	Philips	Neusoft	Siemens	Philips	Neusoft	Siemens	Philips	Neusoft
Mode	Helical	Helical	Axial	Helical	Helical	Axial	Helical	Helical	Axial
kVp	120	120	120	80	120	120	120	120	120
mA	230.25	97.5	126.0	288.0	108.25	200.0	106.75	136.0	375.0
Time (msec)	1000.0	1279.0	3062.5	500.0	438.0	1365.0	500.0	426.25	1220.0
mAs	418.25	124.75	252	119.75	47.25	120	66.25	58	187.5
Thickness (mm)	5	5	5	2	2	2	2	2	2
FOV (mm)	250	250	227.5	301	350	246.25	300	350	360
SFOV (mm)	500	500	500	500	500	500	500	500	500
Filter	H31s	UB	F20	I31f/3	B	F20	I30f/3	B	F20
Matrix	512	512	512	512	512	512	512	512	512
Pitch	0.55	1.0	0.5	1.2	1.0	1.0	0.8	1.0	1.2
Installation year	2013	2008	2019						

**Table 2:** CTDI_vol_, DLP, and effective dose in clinical routine protocols with three different CT scanners.

Protocols	Siemens SOMATOM Definition Flash			Philips Brilliance iCT			Neusoft NeuViz 128		
	CTDI_vol_ (mGy)			CTDI_vol_ (mGy)			CTDI_vol_ (mGy)		
	Mean ± SD	Median	P75	Mean ± SD	Median	P75	Mean ± SD	Median	P75
Brain	42.57 ± 2.08	42.5	43.71	26.00 ± 1.09	26.60	26.60	26.50 ± 1.13	27.30	27.30
Chest	4.48 ± 0.76	4.48	5.12	4.73 ± 0.80	4.52	5.12	7.87 ± 0.65	7.40	8.30
Abdomen	9.54 ± 0.92	9.69	10.29	8.53 ± 0.64	8.63	8.99	10.79 ± 0.27	10.80	10.80
	DLP (mGy.cm)			DLP (mGy.cm)			DLP (mGy.cm)		
Brain	755.17 ± 50.49	764.00	796.50	533.20 ± 25.54	533.80	553.80	505.06 ± 28.22	511.20	524.90
Chest	206.20 ± 59.20	202.50	246.75	182.97 ± 29.67	174.65	199.90	288.71 ± 27.76	294.45	298.80
Abdomen	446.04 ± 49.51	446.15	468.88	479.44 ± 39.27	484.70	513.88	585.91 ± 66.15	571.26	616.95
	Effective dose (mSv)			Effective dose (mSv)			Effective dose (mSv)		
Brain	1.59 ± 0.11	1.60	1.67	1.12 ± 0.05	1.12	1.16	1.06 ± 0.06	1.07	1.10
Chest	2.89 ± 0.83	2.84	3.45	2.56 ± 0.42	2.45	2.80	4.04 ± 0.39	4.12	4.18
Abdomen	6.69 ± 0.74	6.70	7.03	7.19 ± 0.59	7.27	7.71	8.79 ± 0.99	8.57	9.25

**Table 3:** Comparison of local CTDIvol in clinical routine protocols with three different CT scanners with Thailand DRLs, Japan DRLs, ACR DRLs 2018 and ICRP DRLs 2001.

Protocol			CTDI_vol_				
	Siemens SOMATOM Definition Flash	Philips Brilliance iCT	Neusoft NeuViz 128	Thailand DRLs 2021 [15]	Japan DRLs 2020 [16]	ACR DRLs 2018 [17]	ICRP DRLs 2001 [18]
	(Median)	(Median)	(Median)				
Brain	42.5	26.60	27.30	62	77	56	60
Chest	4.48	4.52	7.40	18	13	12	30
Abdomen	9.69	8.63	10.80	18	18	16	35

**Table 4:** Comparison of local DLP in clinical routine protocols with three different CT scanners with Thailand DRLs, Japan DRLs, ACR DRLs 2018 and ICRP DRLs 2001.

Protocol			DLP				
	Siemens SOMATOM Definition Flash	Philips Brilliance iCT	Neusoft NeuViz 128	Thailand DRLs 2021	Japan DRLs 2020	ACR DRLs 2018	ICRP DRLs 2001
	(Median)	(Median)	(Median)				
Brain	764.00	533.80	511.20	1028	1350	962	1050
Chest	202.50	174.65	294.45	417	510	443	650
Abdomen	446.15	484.70	571.26	717	880	781	800

**Table 5:** Comparison of local effective dose in clinical routine protocols with three different CT scanners with R Smith-Bindman and Ioannis Pantos.

Protocol	Effective Dose (mSv)				
	Siemens SOMATOM Definition Flash	Philips Brilliance iCT	Neusoft NeuViz 128	R Smith-Bindman [19]	Ioannis Pantos [20]
	(Median)	(Median)	(Median)		
Brain	1.60	1.12	1.07	2	1.9
Chest	2.84	2.45	4.12	9	7.0
Abdomen	6.70	7.27	8.57	10	7.6

**Table 6:** The modulation transfer function (MTF) of three clinical routine protocols with various CT scanners.

	Brain			Chest			Abdomen		
MTF	Siemens (cy/cm)	Philips (cy/cm)	Neusoft (cy/cm)	Siemens (cy/cm)	Philips (cy/cm)	Neusoft (cy/cm)	Siemens (cy/cm)	Philips (cy/cm)	Neusoft (cy/cm)
0.5	3.12	3.59	4.18	3.68	3.61	3.27	3.43	3.57	3.40
0.1	6.42	5.68	6.91	6.10	5.85	6.03	5.60	5.92	6.21
0.02	8.11	6.80	8.63	7.12	6.86	7.70	6.42	7.19	7.19

**Table 7:** The image noise and uniformity of three clinical routine protocols with various CT scanners.

Protocols	Siemens SOMATOM Definition Flash		Philips Brilliance iCT		Neusoft NeuViz 128		Tolerance
	Mean	SD	Mean	SD	Mean	SD	
Brain							
Noise	3.50	0.00	6.73	0.06	4.63	0.06	
Uniformity	7.00	0.00	7.83	0.15	4.83	0.64	≤5.0
Chest							
Noise	12.10	0.14	19.45	0.07	8.75	0.07	
Uniformity	1.43	0.06	5.80	0.00	7.13	0.06	≤5.0
Abdomen							
Noise	8.00	0.01	19.75	0.07	7.65	0.07	
Uniformity	1.00	0.06	5.40	0.61	7.43	0.06	≤5.0

**Table 8:** The peak NPS frequency and average frequency of three clinical routine protocols with various CT scanners.

		Brain			Chest			Abdomen	
	Siemens	Philips	Neusoft	Siemens	Philips	Neusoft	Siemens	Philips	Neusoft
	(1/mm)	(1/mm)	(1/mm)	(1/mm)	(1/mm)	(1/mm)	(1/mm)	(1/mm)	(1/mm)
Peak NPS Frequency	0.05	0.05	0.05	0.15	0.20	0.05	0.15	0.20	0.05
Average Frequency	0.20	0.23	0.31	0.28	0.29	0.29	0.24	0.29	0.29

## Data Availability

Data are contained within the article.

## Abbreviations

DLP, dose length product; CTDIvol, volume computed tomography dose index; CNR, contrast-to-noise ratio; MTF, modulation transfer function; HCR, high-contrast resolution; LCD, low-contrast detectability; NPS, noise power spectrum.
